# UPLC-ESI-Q-TOF-MS-Based Metabolite Profiling, Antioxidant and Anti-Inflammatory Properties of Different Organ Extracts of *Abeliophyllum distichum*

**DOI:** 10.3390/antiox10010070

**Published:** 2021-01-07

**Authors:** Tong-Kewn Yoo, Won Tae Jeong, Jun Gu Kim, Hyo Seong Ji, Min-A Ahn, Jong-Wook Chung, Heung Bin Lim, Tae Kyung Hyun

**Affiliations:** Department of Industrial Plant Science and Technology, College of Agricultural, Life and Environmental Sciences, Chungbuk National University, Cheongju 28644, Korea; ydk31197586@chungbuk.ac.kr (T.-K.Y.); shewaspretty@chungbuk.ac.kr (W.T.J.); wnsrndlsn@chungbuk.ac.kr (J.G.K.); wlgytjd007@naver.com (H.S.J.); koala0523@chungbuk.ac.kr (M.-A.A.); jwchung73@chungbuk.ac.kr (J.-W.C.)

**Keywords:** *Abeliophyllum distichum*, antioxidant, anti-inflammation, metabolomic profiling, reactive oxygen species

## Abstract

Plant extracts have gained more attention as natural therapeutic agents against inflammation characterized by an overproduction of several inflammatory mediators such as reactive oxygen species and pro-inflammatory cytokines. Although *Abeliophyllum distichum* Nakai is generally known for its ornamental value, recent pharmacological research has demonstrated its potential therapeutic properties. Thus, to further evaluate the applicability of *A. distichum* in the food, cosmetic, and medical industries, we identified the phytochemicals in three organ extracts (fruits: AF, branches: AB, leaves: AL) of *A*. *distichum* and determined their antioxidant and anti-inflammatory activities. Using UPLC-ESI-Q-TOF-MS, a total of 19 compounds, including dendromoniliside D, forsythoside B, isoacteoside, isomucronulatol 7-O-Glucoside, plantamajoside, and wighteone were identified in the *A. distichum* organ extracts. AB exhibited a strong reducing power, an oxygen radical antioxidant capacity, and radical scavenging values compared with other samples, whereas AL exhibited the best anti-inflammatory properties. Gene expression, western blot, and molecular docking analyses suggested that the anti-inflammatory effect of AL was mediated by its ability to suppress lipopolysaccharide (LPS)-induced production of reactive oxygen species and/or inhibit LPS-stimulated activation of extracellular signal-regulated protein kinases (ERK1/2) in RAW264.7 cells. Collectively, these results indicate that AL is a potential source of phytochemicals that could be used to treat inflammation-associated diseases.

## 1. Introduction

Inflammation is the response of the immune system against tissue injury caused by mechanical trauma, bacterial infections, chemical insult, burns, or other harmful agents [1]. Uncontrolled inflammation can lead to tissue and cell damage, chronic inflammation, neoplastic transformation, and is an important factor that influences the development of various chronic diseases [1]. Although the inhibitors of phospholipase A2 (PLA2) and cyclooxygenases (COX) such as steroidal anti-inflammatory drugs (SAIDs) and nonsteroidal anti-inflammatory drugs (NSAIDs) are widely used to treat many inflammatory diseases [2,3], the therapeutic benefits of these drugs are limited by their adverse side effects, which are caused by drug resistance or non-selective inhibition [2,3,4]. 

In addition to their therapeutic benefits, many plants have been found to possess therapeutic compounds, and therefore, have become a major source of novel active compounds and precursors for the synthesis of novel drugs. In fact, approximately 50% of drugs derive directly or indirectly from plant species and their preparations [5]. For example, shikimic acid (3,4,5-trihydroxy-1-cyclohexene-1-carboxylic acid) has been used as a chemical precursor for the industrial synthesis of antiviral drugs Tamiflu (oseltamivir) [6,7]. Additionally, the anti-inflammatory drug Acheflan^®^, which is produced from *Codia verbenacea* essential oil, is another example of a plant-derived drug [8]. Importantly, several medicinal plants have been reported to contain natural anti-inflammatory compounds, and therefore could be used as alternative natural anti-inflammatory agents with minimal side effects [9]. 

*Abeliophyllum distichum* Nakai (common name white forsythia) is a deciduous flowering plant belonging to the Oleaceae family, which contains 24 genera and 688 species [10]. This plant belongs to a monotypic genus (i.e., a genus with only a single species), which highlights the importance of this plant as a unique genetic resource [11]. Although this plant has been cultivated as an ornamental plant in Korea, because of its horticultural value, recent studies have reported that *A. distichum* possesses several therapeutic properties, including antioxidant, anti-inflammatory, anti-melanogenic, anti-cancer, and anti-diabetic effects [11,12,13,14]. Recently, it has been shown that *A*. *distichum* leaf EtOH extract has a higher free radical scavenging activity than its stem extract, suggesting variation in biological activities in different organs of *A*. *distichum* [15]. Among phytochemicals in *A. distichum*, phenolic and phenylethanoid glycosides have been reported as multiple active compounds with therapeutic value [11,14]. For example, acteoside (phenylethanoid glycoside) and isoacteoside (dihydroxypheynylethyl glycoside) identified in leaves of *A. distichum*, exhibited antioxidant and anti-inflammatory activities, respectively [15,16], indicating that *A. distichum* could have functional applications in the cosmetic, biomedical, pharmaceutical, and other industries. While various studies have characterized the phenolic and phenylethanoid glycoside compounds from *A. distichum*, systematic investigation including the comparison of the metabolite composition in different organ and molecular mechanism underlying the anti-inflammatory effects is extremely limited.

Therefore, our study investigated and compared the antioxidant and anti-inflammatory activities of extracts obtained from leaves, branches, and fruits of *A. distichum*. In lipopolysaccharide (LPS)-stimulated RAW264.7 macrophage cells, to investigate the role of *A. distichum* extracts in oxidative stress-induced inflammation, we analyzed the expression pattern of genes, NADPH oxidases (NOX1, NOX2), superoxide dismutase (SOD), catalase (CAT), NAD(P)H quinone oxidoreductase 1 (NQO1), and γ-glutamyl cysteine synthetase (GCLC), involved in reactive oxygen species (ROS) production or antioxidant systems. Additionally, to better understand the antioxidant and anti-inflammatory activities of *A. distichum* extracts, we performed metabolic analysis using ultra-performance liquid chromatography coupled with electrospray ionization-quadrupole-time of flight-mass spectrometry (UPLC-ESI-Q-TOF-MS). 

## 2. Materials and Methods

### 2.1. Preparation and UPLC-ESI-Q-TOF-MS-Based Metabolomic Analysis of A. distichum Organ Extracts 

Three different organs from *A. distichum* obtained from the Chungbuk National University research forest were lyophilized and ground into fine powder. The obtained powder (200 g) were extracted in methanol (MeOH, 1 L) for 24 h at room temperature. After filtration, the MeOH extracts of fruits (AF), branches (AB) and leaves (AL) were concentrated under vacuum in rotary evaporator at 40 °C, as described by Yoo et al. [14]. To investigate the antioxidant and anti-inflammatory activities, the 100 mg of each extract was dissolved in 1 mL dimethyl sulfoxide (DMSO). 

For metabolic analysis, the samples were analyzed using ACQUITY UPLC system (Waters, Milford, MA, USA). Chromatographic separation was performed using a Waters ACQUITY UPLC column (BEH C18: 2.1 × 50 mm, 1.7 μm) at 35 °C with a 0.3 mL/min flow rate. A gradient elution was achieved using two mobile phases consisting of 0.1% (*v*/*v*) aqueous formic acid (mobile phase A) and acetonitrile (mobile phase B). Mass spectrometric analyses were performed using a Xevo G2 Q-TOF LC/MS mass spectrometer system (Waters, Milford, MA, USA) equipped with an ESI source in negative ion mode. The optimized parameters for the mass spectrometric analysis were the following: capillary voltage, 2.5 kV; cone voltage, 25 V; cone gas flow rate, 50 L/h; desolvation gas flow rate, 800 L/h; desolvation gas temp, 350 °C; source temperature 150 °C; collision energies, 30–35 eV. The mass range was set to 50–1200 *m*/*z*. All UPLC-ESI-Q-TOF-MS data were analyzed using the UNIFI software (ver. 1.8) and the ChemSpider database (http://www.chemspider.com/). Resolved peaks were further identified based on reported values from the literature.

### 2.2. Determination of Antioxidant Properties by Chemical-Based Assays 

Reducing power was determined by monitoring Fe^3+^–Fe^2+^ transformation at 750 nm, and the oxygen radical antioxidant capacity (ORAC) value were determined by monitoring the inhibition of peroxyl radical-induced oxidation, as described by Choi et al. [17]. For reducing power assay, ascorbic acid (ASA) was used as a positive control. In addition, the area under the curve of the fluorescence decay curve was used to quantify the ORAC values, and the value was expressed as μM of Trolox equivalents (μM TE). To optimize the concentrations of extracts for antioxidant assays, we tested various concentration (0 to 400 μg/mL) of each extract, and presented data, which exhibited significantly different antioxidant activities between extracts.

The free radical scavenging activity of each extract was analyzed using 1,1-diphenyl-2-picrylhydrazyl (DPPH) and 2,20-azino-bis(3-ethylbenzothiazoline-6-sulfonic acid) (ABTS^•+^). To generate ABTS^•+^, 7 mM ABTS was mixed with 2.45 mM potassium persulfate, and incubated for 16 h in the dark. A total of 180 µL of DPPH solution (0.4 mM in 80% MeOH) or diluted ABTS^•+^ solution was plated in 96 well plates, and 20 µL of each extract (10 mg/mL) was added, followed by serial dilution to each well. The absorbance values were measured at 520 nm (for DPPH redical) or 734 nm (ABTS^•+^), and the RC_50_ (50% reduction of radicals) was determined using the graph plotted inhibition percentage against extract concentration. Butylated hydroxytoluene (BHT) was used as a positive control.

For hydroxyl radical scavenging assay, 100 μg/mL of each extract was mixed with 1 mL reaction buffer (2.8 mM 2-deoxy-2-ribose, 20 mM KH_2_PO_4_-KOH (pH 7.4), 100 μM FeCl_3_, 100 μM EDTA, 1.0 mM H_2_O_2_, and 100 μM ascorbic acid). After incubation for 1 h at 37 °C, 0.5 mL 2.8% TCA and 0.5 mL 1% aqueous TBA was added to the reaction mixtures. The absorbance was measured at 532 nm, after incubation for 30 min at 90 °C. Hydroxyl radical scavenging activity was calculated as described by Lee et al. [15].

### 2.3. Analysis of Nitric Oxide (NO) Production and Cell Viability

Lipopolysaccharide (LPS)-stimulated macrophage is a commonly used model to study the inflammation. To investigate the anti-inflammatory effect of *A. distichum* extracts, RAW264.7 murine macrophage cells (ATCC^®^ TIB-71^TM^, ATCC, Rockville, MD, USA) were cultivated with each extract in the presence of 1 μg/mL LPS. After incubation for 24 h, the cultured medium was used to determine LPS-induced NO production using the Griess reagent and cell viability tests were conducted using MTT assay, as described by Choi et al. [11]. NO concentration was calculated based on a standard curve generated with sodium nitrite. For MTT assay, the resulting formazan crystals were dissolved in DMSO, and optical density was measured at 520 nm.

### 2.4. DCFH-DA (Dichlorofluorescein Diacetate) Assay 

In the RAW264.7 cells, the level of intracellular ROS induced by LPS was detected using the DCFH-DA assay, as described by Choi et al. [11]. RAW264.7 cells were cultivated with or without AL in the presence of LPS. After incubation for 5 h, the cells were washed twice with Hank’s buffered salt solution (HBSS) and incubated with 20 μM of fresh DCFH-DA in HBSS for 30 min at 37 °C in a 5% CO_2_ incubator. DCF fluorescence was determined with fluorescence microplate reader (485 nm excitation and 525 nm emission).

### 2.5. Western Blot 

Equal amounts of proteins extracted from RAW264.7 cells using the RIPA lysis buffer were separated with SDS-PAGE (10% gel) and transferred onto the PVDF membrane [14]. After blocking for 1 h with a 5% skim milk, the target proteins were detected using primary (iNOS, MEK1/2, p-MEK1/2, ERK1/2, p-ERK1/2, and β-actin) and HRP-conjugated secondary antibodies. The proteins were then visualized using a chemiluminescence system.

### 2.6. Quantitative Real-Time PCR (qRT-PCR) 

The isolation of total RNA and cDNA synthesis were performed as described by Yoo et al. [14]. The expression patterns of each gene were analyzed using the Real-time PCR system (Bio-Rad, Hercules, CA, USA). The transcription levels of each gene (*iNOS*, *COX-2*, *IL-6*, *NOX1*, *NOX2*, *SOD*, *CAT*, *NQO-1*, and *GCLC*) were normalized to that of *β-actin*, which was used as an internal standard. The specific qRT-PCR primer pairs are listed in Table S1.

### 2.7. Statistical Analyses

The significant differences between mock control and treated samples were determined using one-way ANOVAs, Duncan multiple range, and Tukey’s honest significance tests. *p* values < 0.05 were regarded as statistically significant difference.

## 3. Results and Discussion

### 3.1. Identification of the Chemical Composition of A. distichum Organ Extracts Using UPLC-ESI-Q-TOF-MS

Although metabolomic approaches including liquid- and gas-chromatography-mass spectrometry have been used to identify and analyze the metabolites of several plants, UPLC-ESI-Q-TOF-MS is thought to be the most effective tool for the on-line structural elucidation of multiple components in plant extracts due to its mass measurement accuracy, high separation resolution, and excellent sensitivity [18]. To characterize the chemical composition of MeOH extracts obtained from *A. distichum* organs, untargeted qualitative analysis of the extracts was conducted via UPLC-ESI-Q-TOF-MS. The detected molecules were characterized by analyzing their fragmentation, which was obtained via ESI-MS in negative mode (Figure S1) coupled with literature data. As shown in Table 1, a total of 19 compounds including phenylpropanoids, phenolic acids, isoflavones, and terpenoids were identified. D-(−)-quinic acid (1), rutin (7), isoaceoside (12), and asiatic acid (19) were identified in all tested extracts. Isoacteoside (12) has been suggested as a major active compound of *A*. *distichum* leaf [16]. However, we found that isoaceoside (12) exist in leaves, as well as fruits and branches of *A*. *distichum* (Table 1). Moreover, five compounds including dendromoniliside D (2), plantamajoside (4), calceolarioside B (5), isomucronulatol 7-O-glucoside (10), and bulleyanin (17) were only identified in AL, whereas another four compounds were only found in AB. This organ-specific distribution of secondary metabolites was likely due to organ-specific metabolic shifts. In Korean black raspberry (*Rubus coreanus* Miquel), the flavonoid biosynthesis process shifts from proanthocyanidins to anthocyanins during the ripening process [19]. Similarly, organ-specific flavonoid metabolic shifts have been reported in various *Scutellaria baicalensis* organs [20]. Alternatively, metabolite production/modification may also be organ-specific. For instance, in *Achyranthes bidentata*, saponins are mainly synthesized in leaves, and transported and modified in roots [21]. The differential mRNA levels of genes involved in the biosynthesis of specific metabolites in different organs also supported the notion that metabolite production/modification is often organ-specific [17,22]. Therefore, differences in phytochemical compositions may affect the biological activities of different *A. distichum* organ extracts.

### 3.2. In-Vitro Antioxidant Properties of A. distichum Organ Extracts

**Figure 1:**
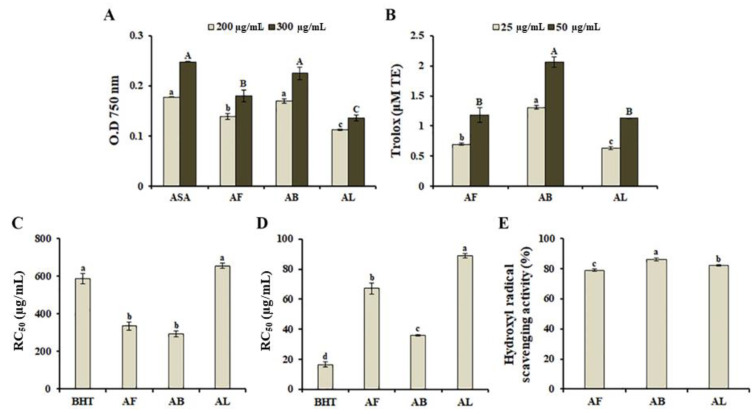
Antioxidant activities of methanol extracts obtained from *Abeliophyllum distichum* organs. Antioxidant activities were analyzed using the reducing power assay (**A**), the ORAC assay (**B**), DPPH free radical scavenging activity (**C**), ABTS cation radical scavenging activity (**D**), and hydroxyl radical scavenging activity (**E**). The ORAC value of each sample is expressed as micromol of Trolox (TE) equivalents. Each value represents the mean ± SE of triplicate measurements. Different superscripted letters are used to indicate the significant differences (*p* < 0.05). Leaf extract, AL; fruit extract, AF; branch extract, AB; ascorbic acid, ASA.

Oxidative stress, which results from oxidant-antioxidant imbalance, deregulates a series of cellular functions, and causes various pathological conditions such as chronic inflammation, aging, asthma, carcinogenesis, cardiovascular dysfunction, diabetes, and neurodegenerative diseases [23]. Therefore, the development of alternative natural antioxidant agents with little or no side effects has attracted considerable attention. To characterize the antioxidant properties of MeOH extracts obtained from *A. distichum* organs, we tested the antioxidant activities of different samples using the reducing power assay and ORAC assay, which are based on single-electron transfer and hydrogen atom transfer reactions, respectively. As shown in Figure 1A, antioxidant activity varied among the different organ extracts used in this study. AB (OD_750_ value = 0.23) exhibited the highest reducing potential, followed by AF (OD_750_ value = 0.18). Similarly, based on the ORAC assay, AB extracts exerted the highest inhibitory effect on peroxyl radical-induced oxidation (Figure 1B). In addition, AB displayed a stronger ability to decrease DPPH free radicals (RC_50_ = 294.5 ± 16.4 µg/mL, Figure 1C), ABTS cation radicals (RC_50_ = 36.0 ± 0.63 µg/mL, Figure 1D), and hydroxyl radical scavenging activity (86 ± 0.97%) than other organ extracts, suggesting that *A. distichum* branches possess a stronger antioxidant activity than that of the other analyzed organs. Recently, it has been shown that antioxidant activity of *A. distichum* leaf EtOH extracts is slightly higher than *A. distichum* stem EtOH extracts [15]. The variation of antioxidant activity might be mediated by phytochemical compositions and concentrations, which are influenced by season, climatic conditions, stages of maturity, and extraction conditions.

Plant-derived polyphenolic compounds possess several beneficial biological properties, including antioxidant, antibacterial, anti-inflammatory, anti-cancer, and anti-diabetic activities [24]. In *A. distichum* organ extracts, AB contained the highest amount of phenolic compounds (59.8 ± 1.34 μg/mg GAE) but also exhibited the lowest total flavonoid content (14.5 ± 1.71 μg/mg QE) among the examined extracts (Figure S2). Additionally, various polyphenolic compounds including α-glucosyl hesperidin (3), and wighteone (16) were identified in AB (Table 1). Among them, wighteone (16), which has been isolated from *Cudrania fruticosa* root [25], whole-plant extracts of *Lupinus texensis* [26], *Ficus tikoua* root [27], and *Erythrina stricta* stem bark [28], exhibited antimicrobial and antioxidant activities [26,28]. Collectively, these findings indicate that differences in the antioxidant activities of organ extracts are likely mediated by differences in the compositions and concentrations of active compounds such as polyphenols.

### 3.3. Effects of A. distichum Organ Extracts on LPS-Induced NO Production

**Figure 2:**
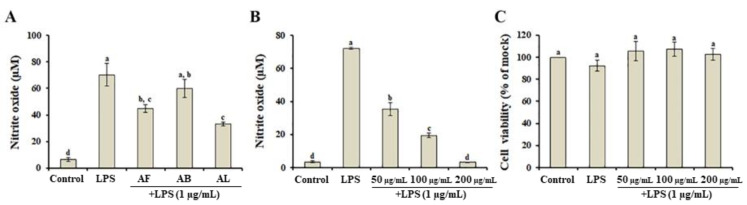
Anti-inflammatory effect of *Abeliophyllum distichum* organ extracts in LPS-treated RAW264.7 macrophages. (**A**) Effect of *A. distichum* organ extracts on LPS-induced NO production. Dose-dependent effect of *A. distichum* leaf extract on LPS-induced NO production (**B**) and cytotoxicity (**C**) in RAW264.7 macrophages. Mean ± SE with different letters indicate the significant differences (*p* < 0.05). Leaf extract, AL; fruit extract, AF; branch extract, AB.

RAW264.7 cells, a monocyte/macrophage-like cell line, stimulated with LPS (i.e., a potent inducer of inflammation) are commonly used to screen for anti-inflammatory properties in plant extracts by measuring NO production. Therefore, to investigate whether *A. distichum* organ extracts possessed anti-inflammatory activity, the inhibitory effects of each organ extract on LPS-stimulated NO production in RAW264.7 cells were examined. As shown in Figure 2A, LPS significantly increased NO production by approximately 11-fold compared to LPS-untreated cells, while 50 μg/mL of AL suppressed LPS-induced NO production by more than 50% beyond the level of the DMSO-treated mock control. Additionally, AL treatment markedly decreased LPS-induced NO increases in a dose-dependent manner (Figure 2B). To assess whether AL affected cell viability, RAW264.7 cells were incubated with LPS in the presence of AL. Our results suggest that AL did not exhibit the cytotoxicity regardless of the presence of LPS (Figure 2C), suggesting that the inhibition of LPS-induced NO production by AL was not mediated by RAW264.7 cell viability.

**Figure 3:**
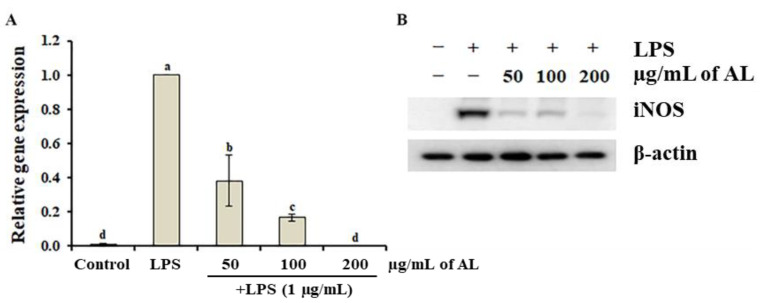
Effect of *Abeliophyllum distichum* leaf extract on the expression level (**A**), and protein level (**B**) of iNOS in LPS-treated RAW264.7 macrophages. The transcription and translation levels of iNOS were investigated using qRT-PCR (**A**) and western blot (**B**). All data are reported as mean ± SE. Different superscripted letters are used to indicate the significant differences (*p* < 0.05).

In macrophage cells, NO is produced by NO synthase (NOS), which is further divided into three classes: endothelial NOS (eNOS), neuronal NOS (nNOS), and inducible NO synthase (iNOS). eNOS and nNOS are known to be constitutively expressed, whereas iNOS is induced by inflammatory stimuli [29]. This suggests that LPS-induced NO production is mainly mediated by iNOS induction [30], indicating that the suppression of excessive iNOS-derived NO production can serve as a promising strategy to the treatment of inflammatory disorders. Thus, to investigate whether the inhibitory effect of AL on LPS-induced NO production was mediated by the suppression of iNOS, LPS-induced iNOS transcription and translation were determined in AL-treated RAW264.7 cells. LPS treatment significantly induced *iNOS* transcription within 24 h compared to non-treated cells. However, this expression level of LPS-induced *iNOS* was decreased by AL treatment (Figure 3A). Consistently with these findings, Western blot analyses elucidated an increase in iNOS protein expression in response to LPS, which was almost completely inhibited by AL (Figure 3B), suggesting that the inhibition of LPS-induced NO production by AL treatment was due to the inhibition of *iNOS* expression. iNOS-generated NO reportedly interacts with mitochondrial cytochrome *c* oxidase and competes with oxygen, resulting in a decrease in adenosine triphosphate production [31,32]. Therefore, the inhibitory effect of AL on iNOS-derived NO production in LPS-stimulated cells suggested that AL could be used as a plant-derived drug for the removal of cytotoxic NO.

**Figure 4:**
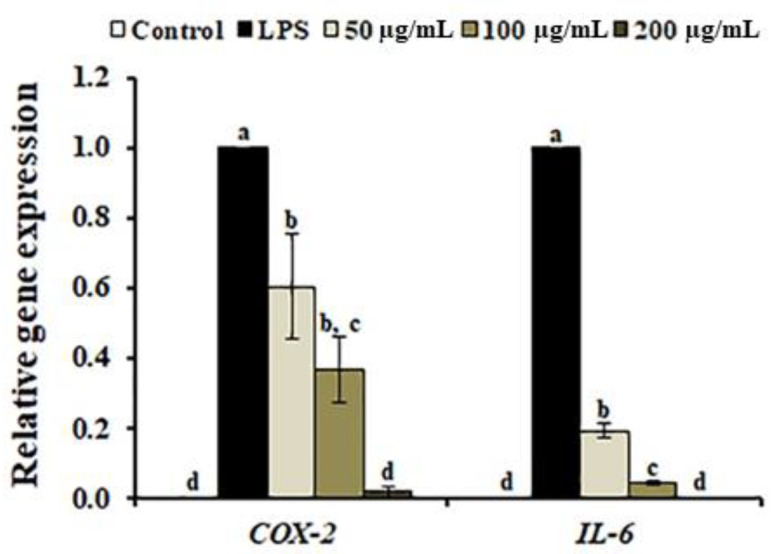
Effect of *Abeliophyllum distichum* leaf extract on *COX-2* and *IL-6* expression in LPS-treated RAW264.7 macrophages. Gene transcript levels were normalized to *β-actin* and were expressed relative to the value of LPS alone. Different superscripted letters indicate the significant differences (*p* < 0.05).

In LPS-stimulated macrophages, inflammatory signaling is induced by the pro-inflammatory gene expression, resulting in the generation of pro-inflammatory mediators including NO, cyclooxygenase-2 (*COX-2*), iNOS, interleukin 6 (IL-6), and prostaglandin (PGE_2_) [33]. Therefore, many phytochemicals and plant extracts capable of reducing or inhibiting the production of pro-inflammatory mediators has been suggested as potential anti-inflammatory agents. To further analyze the anti-inflammatory properties of AL, we determined the expression levels of *IL-6*, and *COX-2*. As shown in Figure 4, LPS-induced expression of *IL-6*, and *COX-2* was significantly decreased by AL in a dose-independent manner. Among the metabolic compounds identified in AL, plantamajoside (4) is known to exert anti-inflammatory effects via the inhibition of LPS-induced IL-6 production [34]. Additionally, calceolarioside B (5), phenylethanoid glycoside, has been exhibited to inhibit TNF-α-induced IL-6 production in MG-63 cells [35]. Furthermore, rutin (7) has been suggested as a candidate therapeutic agent for treatment of inflammatory diseases through inhibition of the high mobility group box 1-stimulated production of IL-6 [36], isoacteoside (12) significantly inhibited the production of phorbol 12-myristate 13-acetate and calcium ionophore A23187 (PMACI)-induced pro-inflammatory mediators in HMC-1 cells [16]. Therefore, the aforementioned compounds are likely responsible for the anti-inflammatory property of AL, suggesting that the anti-inflammatory activity of AL should be modulated by the suppression of pro-inflammatory mediators and cytokine production.

### 3.4. Effects of A. distichum Leaf Extract on LPS-Induced ROS Production in RAW264.7 Cells

**Figure 5:**
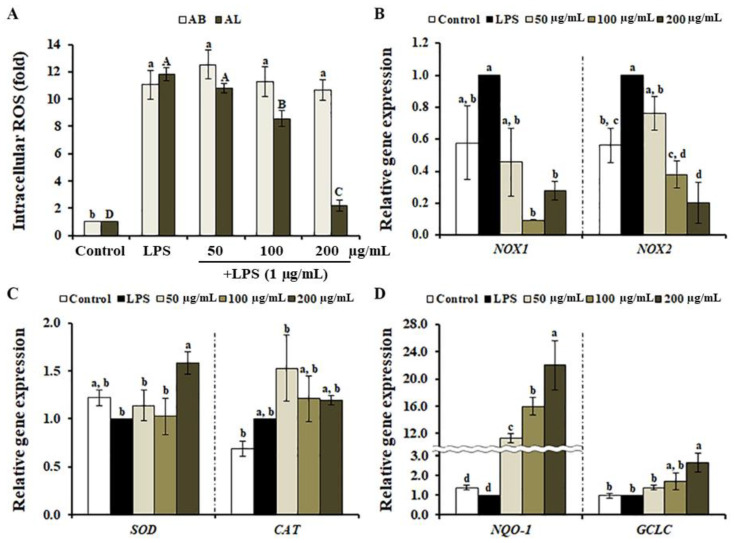
Effects of *Abeliophyllum distichum* leaf extract on LPS-induced ROS production in RAW264.7 macrophages. The inhibitory effect of *A. distichum* leaf (AL) or branch (AB) extract on LPS-induced ROS production was determined using the DCFH-DA (**A**). LPS-treated RAW264.7 cells in the presence of AL were used to analyze the expression level of genes involved in ROS production (**B**), cellular oxygen metabolism (**C**), and Nrf2-targeted antioxidant and detoxification (**D**). The transcript levels of each gene was normalized to *β-actin* and expressed relative to the value of LPS alone. All data were reported as the mean ± SE. Different superscripted letters indicate the significant differences (*p* < 0.05).

In macrophages, LPS triggers the ROS production through the induction of NOXs and the inhibition of the antioxidant system [37,38], suggesting that the inhibition of ROS formation via the suppression of genes involved in ROS production, and/or enhancement of antioxidant mechanisms are all potential targets for the treatment of inflammatory disorders [39]. To analyze the effect of *A. distichum* organ extracts on LPS-induced ROS production in RAW264.7 cells, we determined the level of intracellular ROS using the fluorescent probe DCFH-DA. When RAW264.7 cells were treated with AL, the production of LPS-induced ROS was significantly suppressed in a dose-dependent manner (Figure 5A). Interestingly, AB did not inhibit LPS-induced ROS production, despite exhibiting strong antioxidant properties in our chemical-based assays (Figure 1). DCFH-DA is a common reagent used to detect intracellular ROS levels via the oxidation of DCFH to fluorescent DCF. LPS induced the production of hydroxyl radicals, hydrogen peroxide, and peroxinitrite, resulting in the induction of DCFH oxidation [40]. This reaction is inhibited when bioactive compounds stimulate the intracellular antioxidant defense system or act as ROS-scavengers once the cell membrane is compromised/breached [11,41]. Therefore, one possible explanation for the lack of inhibitory effects of AB on LPS-induced ROS levels might be the inability of the bioactive compounds in AB to penetrate weak cell membranes. To further characterize the inhibitory effect of AL on LPS-induced ROS production, we determined the transcription levels of genes involved in ROS generation and the antioxidant system. As shown in Figure 5B, the expression of LPS-induced *NOX-1* and *NOX-2*, which are involved in LPS-induced oxidative stress [42], was inhibited by AL in a dose-independent manner, suggesting that AL suppressed LPS-induced ROS production via down-regulation of *NOX-1* and *NOX-2*. However, AL did not significantly change the expression of *SOD* and *CAT*, important enzymes in cellular oxygen metabolism, in LPS-stimulated RAW264.7 cells (Figure 5C). In macrophages, the activation of nuclear factor erythroid 2-related factor 2 (Nrf2) plays an important role in cellular redox homeostasis via the induction of antioxidant and detoxifying enzymes [43]. In AL-treated RAW264.7 cells, we found the increasing expression levels of Nrf2-targeted antioxidant and detoxification enzymes, including *NQO1* and *GCLC* (Figure 5D). Recently, it has been shown that plantamajoside (2) protects rat cardiomyocytes H9c2 cells against hypoxia/reoxygenation-induced injury via the enhancement of Nrf2-targeted antioxidant and detoxification pathways [44]. Although bioactive compounds in AL may also scavenge LPS-induced ROS, this finding suggests that the suppression of LPS-induced ROS by AL is likely mediated by either the inhibition of ROS production or the enhancement of the antioxidant system.

### 3.5. Effects of A. distichum Leaf Extract on the LPS-Induced MEK-ERK1/2 Pathway in RAW264.7 Cells

**Figure 6:**
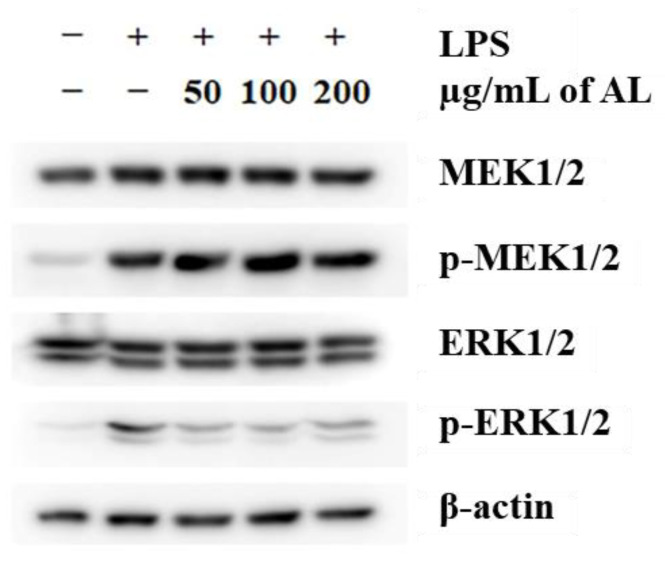
Inhibition of ERK1/2 activation by *A. distichum* leaf extract in LPS-treated RAW264.7 macrophages. The activation of MEK1/2 and ERK 1/2 was analyzed by western blot.

Mitogen-activated protein kinases (MAPKs), including ERK1/2, JNK, and p38, are well-characterized serine/threonine protein kinases that regulate fundamental biological processes and cellular responses such as proliferation, inflammation, apoptosis, and tumorigenesis [45]. In LPS-stimulated cells, MAPKs are activated either by the activation of signaling pathways or by LPS-induced ROS production [46,47]. The inhibition of MEK1/2 by the selective MEK inhibitor U0126 has been exhibited to inhibit the LPS-mediated induction of several inflammatory cytokines and PGE_2_ [48]. Additionally, the LPS-induced MEK/ERK1/2 pathway was inactivated by treatment with an extract obtained from the aerial (i.e., above-ground) structures of *A. distichum* [11]. Based on these findings, we hypothesized that AL regulates the LPS-MEK/ERK1/2 pathway in macrophages, although multiple pathways are activated in response to LPS. To test this hypothesis, we analyzed the inhibitory effect of AL on LPS-induced MEK/ERK1/2 activation. As shown in Figure 6, LPS treatment led to a dynamic activation of MEK1/2 and ERK1/2. Surprisingly, AL treatment was not enough to suppress the phosphorylation of MEK1/2, indicating that the anti-inflammatory effect of AL is not mediated by the inactivation of toll-like receptor 4 (TLR4)-dependent MEK signaling. However, LPS-induced activation of ERK1/2 was strongly inhibited by AL, and this inactivation was not mediated by the inhibition of *de novo* ERK1/2 protein synthesis and MEK1/2 activation. As described above, the inhibition of MEK1/2 activity suppressed LPS-induced inflammation [48]. Based on our analysis of the interaction between compounds in AL and MEK1/2 using a structure-based molecular docking approach, we found that dendromoniliside D (2) (−5.71 ± 0.15 kcal/mol) and isomucronulatol 7-O-glucoside (10) (−6.75 ± 0.38 kcal/mol) in AL had a higher binding affinity to MEK1 than U0126 (−5.32 ± 0.14 kcal/mol) (Table S2), indicating that the inactivation ERK1/2 was due to the inhibition of MEK activity via the bioactive compounds in AL. The MAPK phosphatases (MKPs) or dual-specificity phosphatases play as key negative regulators of MAPK cascades [49]. It has been shown that malvidin (i.e., a polyphenolic compound)-induced MKP-1 transcription is required for the suppression of LPS-induced TLR4 signaling [50]. Although further research is required to analyze the effect of AL on MPK induction, our findings indicated that inactivation of ERK1/2 by AL-induced MKPs led to the inhibition of LPS-induced inflammation, among other potential mechanisms.

## 4. Conclusions

To assess the bioactive compounds of *A*. *distichum*, UPLC-ESI-Q-TOF-MS approach was used to obtain a detailed profile of the metabolic compounds in three different organs of *A*. *distichum*. 19 compounds including dendromoniliside D (2), rutin (7), isomucronulatol 7-O-glucoside (10), forsythoside B (11), and isoacteoside (12) were identified in *A*. *distichum* organ extracts. Moreover, we determined the antioxidant and anti-inflammatory activities of *A. distichum* organ extracts. The gene expression analysis indicates that AL suppressed the LPS-induced ROS via the modification of antioxidant system. Furthermore, the molecular docking analysis indicates that isomucronulatol 7-O-glucoside (10) (−6.75 ± 0.38 kcal/mol) identified in AL exhibited good binding affinity to MEK1, suggesting that AL has an inhibitory effect on the LPS-induced pro-inflammatory mediator production through either the suppression of ROS production or the inhibition of ERK1/2 signaling. Collectively, these results indicate that AL contains phytochemicals that could be used to treat and control inflammatory diseases. Further in vivo studies are necessary to assess crucial physiological aspects of *A*. *distichum* extract administration, including its safety, bioavailability, and metabolism.

## Figures and Tables

**Table 1:** Phytochemical constituents identified in *Abeliophyllum distichum* leaf (AL), fruit (AF), and branch (AB) extracts.

Peak No.	Rt (min)	Neutral Mass (Da)	Observed *m*/*z*	Mass Error (mDa)	Formula	Proposed Molecule	Fragment Ions	Organ
1	0.52	192.0634	191.0555	−0.6	C_7_H_12_O_6_	D-(−)-Quinic acid	173.04, 149.04, 127.04, 109.03, 93.03, 85.03	AF, AB, AL
2	5.43	446.2152	445.2074	−0.5	C_21_H_34_O_10_	Dendromoniliside D	401.14, 269.10, 229.07, 185.04, 141.02, 103.04	AL
3	7.02	772.2426	771.2342	−1.1	C_32_H_36_O_22_	α-Glucosyl hesperidin	661.18, 607.19, 591.19, 229.07, 179.03, 161.02	AB
4	7.09	640.2003	639.1922	−0.9	C_29_H_36_O_16_	Plantamajoside	475.14, 185.04, 179.03, 161.02	AL
5	7.37	478.1475	477.1399	−0.4	C_23_H_26_O_11_	Calceolarioside B	221.04, 161.02	AL
6	7.50	786.2582	785.2512	−0.3	C_35_H_46_O_20_	Echinacoside	624.22, 429.15, 387.15, 305.09, 179.03, 161.02, 135.04	AF
7	8.01	610.1534	609.1459	−0.2	C_27_H_30_O_16_	Rutin	301.03, 300.03, 299.02	AF, AB, AL
8	8.14	488.3138	487.3055	−1.0	C_24_H_40_O_10_	Senegenic acid	367.27, 309.22, 290.22, 225.16	AF, AL
9	8.48	462.1526	461.1451	−0.2	C_23_H_26_O_10_	6′-coumaroyl-1′-*O*-[2-(3,4-dihydroxyphenyl)ethyl]-β-d-glucopyranoside	265.07, 205.05, 163.04, 145.03	AF, AL
10	8.56	464.1682	463.1599	−1.1	C_21_H_20_O_12_	Isomucronulatol 7-*O*-Glucoside	341.06, 327.04, 222.09, 141.02, 137.02	AL
11	8.64	756.2477	755.2397	−0.7	C_32_H_38_O_20_	Forsythoside B	725.23, 593.21, 325.09, 275.08, 179.03, 161.03, 135.04	AF, AB
12	8.96	624.2054	623.1979	−0.2	C_29_H_36_O_15_	Isoacteoside	461.16, 179.03, 161.02	AF, AB, AL
13	9.25	520.1945	519.1869	−0.3	C_23_H_22_O_13_	Brusatol	465.18, 447.09, 357.13, 342.11, 209.08, 103.04	AB
14	9.94	374.1366	373.1292	−0.0	C_20_H_22_O_7_	(−)-Nortrachelogenin	343.12, 313.11, 207.07, 181.05, 145.03	AB, AL
15	12.42	358.1416	357.1343	−0.1	C_19_H_18_O_7_	Miroestrol	342.11, 299.09, 195.07, 151.04, 122.04	AB
16	12.72	338.1154	337.1081	−0.0	C_20_H_18_O_5_	Wighteone	295.06, 267.07, 190.06, 175.04, 151.04	AB
17	13.09	534.2465	533.2393	−0.1	C_24_H_22_O_14_	Bulleyanin	383.11, 343.16, 341.14, 163.04, 145.03, 121.03	AL
18	13.98	436.2097	435.2027	−0.2	C_21_H_24_O_10_	Polhovolide	229.07, 227.11, 185.04, 163.00, 141.02	AF, AL
19	20.65	488.3502	487.3425	−0.4	C_30_H_48_O_5_	Asiatic acid	455.32, 409.31	AF, AB, AL

## Data Availability

The data presented in this study are available on request from the corresponding author. The data are not publicly available due to reasons of privacy.

## Supplementary Materials

The following are available online at https://www.mdpi.com/2076-3921/10/1/70/s1, Figure S1: Chromatogram of *A. distichum* organ extracts obtained by UPLC-ESI-Q-TOF-MS in negative mode, Figure S2: Total phenolic content and flavonoid content in *A. distichum* organ extracts, Table S1: Primer sequences used in this study, Table S2: In silico defined molecular docking of some metabolic compounds to MEK1. Supplementary Methods: Determination of total phenolic content (TPC) and total flavonoid content (TFC), Molecular docking analysis.
