# Supplementary material for: UPLC-ESI-Q-TOF-MS-Based Metabolite Profiling, Antioxidant and Anti-Inflammatory Properties of Different Organ Extracts of Abeliophyllum distichum
Source: Antioxidants (Basel). 2021 Jan 7;10(1):70. doi: 10.3390/antiox10010070 (PMC7827262; doi:10.3390/antiox10010070)
Supplement: Supplementary file 1 [file antioxidants-10-00070-s001.zip › antioxidants-1053187-supplementary.doc]

**Supplementary Methods**

1. Determination of total phenolic content (TPC) and total flavonoid content (TFC)

The total phenolic content were measured according to the Folin-Ciocalteu method with gallic acid as the standard. Folin-Ciocalteu reagent (2N, 50 µl) was mixed with 100 µl of each extract. After 5 min incubation at room temperature, 0.3 ml of 20% sodium carbonate was added to each mixture. The absorbance of the reaction mixtures was measured at 725 nm using UV-spectrophotometer. TPC was expressed in microgram of gallic acid equivalents (GAE) per milligram of extract (µg GAE/mg extract).

To analyze TFC of each extract, 100 µl of each extract was mixed with 20 µl of 10% aluminum nitrate, 20 µl of 1 M potassium acetate, and 860 µl of 80% ethanol. The mixtures were incubated at room temperature for 40 min, and then the absorbance was determined at 415 nm. The total flavonoid content was expressed in microgram of quercetin equivalents (QE) per milligram of extract (µg GE/mg extract).

2. Molecular docking analysis

Molecular blind docking and defined docking were performed using MEK1 (PDB code, 3E8N), Dendromoniliside D (PubChem CID, 11015758), Plantamajoside (PubChem CID, 5281788), Calceolarioside B (PubChem CID, 5273567), Isomucronulatol 7-O-glucoside (PubChem CID, 125142), and U0126 (PubChem CID, 3006531). AutoDock4 with Lamarckian Genetic Algorithm was used to calculate the molecular blind and defined docking results, as described by Seo and Efferth [1]. Grid box was created covering whole residues, and docking parameters were set to 100 runs and 2,500,000 energy evaluations for blind docking. For defined docking, grid maps were created using blind docking results, and docking parameters of defined docking were set to 250 runs and 2,500,000 energy evaluations each time. The lowest binding energies and predicted inhibition constants were obtained from the docking log files.

Ref.

[1] Seo, E.J.; Efferth, T. Interaction of antihistaminic drugs with human translationally controlled tumor protein (TCTP) as novel approach for differentiation therapy. *Oncotarget*. **2016**, *7*, 16818-1639.

**Supplementary Tables**

Table S1. Primer sequences for qReal-time PCR analysis.

| **Primer** | **Sequences (5`-3`)** | **Accession number** |
| --- | --- | --- |
| iNOS -F | TCCTACACCACACCAAAC | AF427516.1 |
| iNOS -Rev | CTCCAATCTCTGCCTATCC |  |
| COX-2 -F | CCTCTGCGATGCTCTTCC | AF233596.1 |
| COX-2 -Rev | TCACACTTATACTGGTCAAA |  |
| IL-6 -F | CCACTTCACAAGTCGGAGGC | DQ788722.1 |
| IL-6 -Rev | GTGCATCATCGCTGTTCATA |  |
| NOX1 -F | AAGTGGCTGTACTGGTTGG | NM_172203.2 |
| NOX1 -Rev | GTGAGGAAGAGTCGGTAGTT |  |
| NOX2 -F | ACTTCTTGGGTCAGCACTGG | FJ168469.1 |
| NOX2 -Rev | ATTCCTGTCCAGTTGTCTTC |  |
| SOD -F | CAGCATGGGTTCCACGTCCA | BC032986.1 |
| SOD -Rev | CACATTGGCCACACCGTCCT |  |
| CAT -F | AAGACAATGTCACTCAGGTG | BC013447.1 |
| CAT -Rev | GGCAATGTTCTCACACAGGC |  |
| NQO-1 -F | TATCCTTCCGAGTCATCTCT | NM_008706.5 |
| NQO-1 -Rev | TCTGCAGCTTCCAGCTTCTT |  |
| GCLC -F | CAATGGGAAGGAAGGGGTAT | U85498.1 |
| GCLC -Rev | TCAGGATGGTTTGCAATGAA |  |
| *β*-actin -F | CCCACTCCTAAGAGGAGGAT | NM_007393.5 |
| *β*-actin -Rev | AGGGAGACCAAAGCCTTCAT |  |

Table S2. *In silico* defined molecular docking of some metabolic compounds to MEK1.

| **Protein** | **Compounds** | **Lowest binding energy** **(kcal/mol)** | **Mean binding energy** **(kcal/mol)** | **Residues involved hydrogen bond interaction** | **Residues involved in**  **hydrophobic interaction** | **pKi (mM)** |
| --- | --- | --- | --- | --- | --- | --- |
| MEK1 | Dendromoniliside D | -7.03 ± 0.07 | -5.71 ± 0.15 | GLY79, ARG234 | GLY80, LYS97, HIS188, ARG189, ASP190, ASN195, ASP206, ASP208, PHE209, GLY210, LEU215, ILE216, MET219, TYR229, MET230 | 0.007 ± 0.001 |
|  | Plantamajoside | -4.07 ± 0.11 | -4.07 ± 0.11 | LYS97, HIS145, MET146, SER150, SER194 | LEU74, ALA76, GLY77, VAL82, ALA95, ILE99, MET143, GLY149, ASP152, GLN153, ASP190, LYS192, ASN195, LEU197, ASP208 | 1.081 ± 0.203 |
|  | Calceolarioside B | -4.21 ± 0.07 | -3.55 ± 0.30 | SER212 | GLY77, GLY79, GLY80, LYS97, ILE99, LEU116, VAL127, ILE141, MET143, ASP190, ASP208, PHE209, GLY210, VAL211, LEU215, MET219 | 0.824 ± 0.089 |
|  | Isomucronulatol 7-O-glucoside | -7.85 ± 0.18 | -6.75 ± 0.38 | LYS97, SER194, PHE209 | LEU74, GLY77, GLY79, GLY80, VAL82, ALA95, ILE99, LEU118, VAL127, PHE129, ILE141, MET143, ASP190, LYS192, SER194, ASN195, PHE209, VAL211 | 0.002 ± 0.001 |
|  | U0126 | -6.17 ± 0.10 | -5.32 ± 0.14 | - | GLY79, LYS97, ILE99, HIS188, ARG189, ASP190, ASN195, ASP208, PHE209, GLY210, VAL211, SER212, LEU215, ILE216, MET219, MET230, ARG234 | 0.031 ± 0.005 |

**Supplementary Figures**

Figure S1. Chromatogram of *A. distichum* organ extracts obtained by UPLC-ESI-Q-TOF-MS in negative mode.

Figure S2. Total phenolic content (A) and total flavonoid content (B) in *A. distichum* organ extracts. Values with different superscripted letters are significantly different (*p* < 0.05).
